# Comparing the awareness of and beliefs in sexually transmitted infections among university students in Madagascar and the United States of America

**DOI:** 10.7717/peerj.4362

**Published:** 2018-02-21

**Authors:** Peter R. Reuter, Shannon McGinnis, Kim E. Reuter

**Affiliations:** 1 Marieb College of Health & Human Services, Florida Gulf Coast University, Fort Myers, FL, United States of America; 2 College of Public Health, Temple University, Philadelphia, PA, United States of America; 3 Africa Field Division, Conservation International, Gaborone, Botswana

**Keywords:** Sexual health, Sexually transmitted infections, United States of America, HIV/AIDS, Madagascar, University students

## Abstract

Young adults have a higher risk of contracting sexually transmitted infections (STIs) than other age groups. This risk may be mediated by their social and cultural setting which can impact young adults’ awareness of, beliefs in, and risk of contracting STIs (including HIV/AIDS). In order to understand how these factors vary among young adults of different cultures, it is important to study these issues on a cross-cultural scale. This study aimed to increase understanding of the relationship between the culture of a place of study and: (1) STI awareness; (2) belief in STIs; and (3) self-reported STI prevalence in the study population. Survey data were collected from university students in Madagascar (*n* = 242 surveys in 2013) and the United States of America (*n* = 199 surveys in 2015). Compared to students at the American university, students at the Malagasy university: (1) did not appear to have a conclusively lower awareness of STIs; (2) did not differ in rates of belief in the existence of gonorrhea and syphilis, but had higher rates of disbelief in HIV/AIDS; and (3) were more likely to report having been infected with syphilis and gonorrhea, but not with HIV/AIDS. Students at the Malagasy university also listed different reasons than the students at the American university for why they believed in the existence of STIs. These findings highlight the need for further cross-cultural research to better adapt intervention strategies to different cultural settings.

## Introduction

It is estimated that one million curable cases of STIs are contracted globally each day (Newman et al., 2015). Compared to other age groups, young adults have a higher risk of contracting and spreading STIs and are more likely to engage in high-risk sexual behaviors ( UNICEF/UNAIDS/WHO, 2002; CDC, 2013). Although global statistics on STIs are lacking, young adults are affected by a significant number of the new STI cases each year (Dehne & Riedner, 2001) and in 2014, an estimated four million young people aged 15 to 24 were living with HIV/AIDS (UNAIDS, 2014). Due to the disease burden attributed to STIs and HIV/AIDS, there is a need to improve young adults’ understanding and awareness around these issues globally (MacDonald et al., 1990; UNICEF/ UNAIDS/WHO, 2002; Kurkowski et al., 2012). However, to develop effective and targeted interventions at the local level, it is important to first understand how awareness, beliefs, and disease risks vary in different cultural settings.

An individual’s cultural and social setting can influence their awareness of and beliefs in STIs and HIV/AIDS as well as their disease risk. First, studies have indicated that young adults have low-levels of awareness of STIs globally (including HIV/AIDS; UNAIDS, 2014). This may be influenced by limited access to comprehensive sexual health education due to social stigma (adolescents in Zimbabwe; Sherman & Bassett, 2007) or inability to accurately describe aspects of sexual health due to limited vocabulary available in some cultures (young women aged 16 to 33 in Aboriginal Australian cultures; Ireland et al., (2015). Second, young people have expressed disbelief in the existence of STIs and/or HIV/AIDS in both developed (e.g., within African-American communities in the United States; Bird & Bogart, 2005) and developing countries (e.g., among young people aged 20 to 34 years in Mali; Castle, 2003). Disbelief or skepticism can be due to perceived government discrimination (Bird & Bogart, 2005), lack of perceived STI cases in other areas of the world, and confusion about disease transmission (Castle, 2003). In some cases, highly educated individuals have expressed more disbelief (Castle, 2003). Finally, cultural and social norms impact young adults’ risk of contracting STIs by directly influencing sexual health knowledge and risky sexual behaviors (Sherman & Bassett, 2007) or in some cases by promoting culturally accepted skepticism or disbelief in some STIs (skepticism has increased disease risk, Bird & Bogart, 2005; skepticism is a barrier to HIV/AIDS counseling and testing, Castle, 2003). Varying moral, religious, and social norms influence condom access and use in different cultural settings (reviewed by Sarkar, 2008), and whether young adults receive adequate sexual health education prior to sexual encounters (in Kenya; Juma et al., 2014), both of which influence an individual’s decision-making, behavior, and disease risk.

Given the importance of culture on young adult sexual behavior, it is important to examine how the culture within a place of study—for example where young adults attend university—influence sexual behavior. Cross-cultural studies yield information that is useful in informing public health programming for young adults. For example, a study comparing American and South African university students, found that Americans were more likely to stigmatize gay people and individuals with HIV/AIDS, had higher condom self-efficacy, and a lower perceived risk of infection than South Africans, although both populations engaged in high-risk sexual behavior (Duncan et al., 2005). In another study, a comparison of university students from the United States, Turkey, and South Africa found differences in sexual behaviors (e.g., age of sexual debut, number of partners, condom usage) and perceived HIV/AIDS risk (Adefuye et al., 2011). These findings are important to advise locally relevant interventions or for adapting existing evidence-based interventions to the cultural settings within a place of study.

This study aimed to increase understanding of the influence of cultural setting on young adults’ sexual health by conducting a survey of university students at two different places of study. The survey was conducted with students at a Malagasy university in 2013 (Reuter, Randell & Reuter, 2015) and with students at an American university in 2015. This study used the place of study as a proxy for culture. The objectives of this study were to understand the role of place of study on: (1) young adults’ awareness of STIs; (2) young adults’ belief in certain STIs; and (3) the self-reported prevalence of STIs in the study population (as a measure of disease risk among young adults).

For objective one we hypothesized that: (1) STI awareness would be lower at the Malagasy university than at the American university (based on data indicating that STI knowledge is higher in the USA than other nations, Benotsch et al., 2004, and has been consistently low in Madagascar, Leutscher et al., 2003). For objective two, and lacking information to the contrary in the literature, we hypothesized that (2) belief in the existence of STIs would not differ between the two different places of study. For objective three, we hypothesized that (3) the students at the Malagasy university would have higher rates of self-reported STIs than the students at the American university (due to high rates of STI transmission in Madagascar, Behets et al., 2001; Leutscher et al., 2003; Lanouette et al., 2003; UNICEF/UNAIDS/WHO, 2002). Results include both quantitative and qualitative findings.

### National cultures

In this study, we compare students at a university in northern Madagascar to students in the southern United States of America. Although these students may have not been Malagasy or American, the cultural context of their place of study (i.e., their place of study being either in Madagascar or America) did likely differ due to the different “national cultures” (Dodge et al., 2005) of the two institutions included in this study. Therefore, we provide an overview of the national cultures of Madagascar and the United States of America, below.

#### Madagascar

Although Madagascar is home to various ethnic groups each with their own sub-cultures, nationally, the country is influenced by African and Asian cultures (Bureau of African Affairs, 2011). Traditional religions are common, and slightly less than half of the population practices Christianity (with institutional and traditional religious beliefs not being mutually exclusive; Bureau of African Affairs, 2011). Madagascar is socially conservative, and women often occupy social roles considered inferior to men in traditional society (Chapin Metz, 1994). Poverty rates in the country remain high (The World Bank, 2016) and political challenges have caused large government spending cuts to the health sector in the past (IRIN News, 2012).

Previous studies in Madagascar have found that STI and HIV/AIDS knowledge is low, especially among young adults (Leutscher et al., 2003; Glick, Randriamamonjy & Sahn, 2009). Only 27.1% of young men aged 15–19 (compared to 46.6% of all men sampled) knew that HIV is spread through sexual contact (Leutscher et al., 2003) and older women were more knowledgeable about HIV/AIDS prevention than those in the 15–20 age range (Glick, Randriamamonjy & Sahn, 2009). To our knowledge, previously published data from this survey (Reuter, Randell & Reuter, 2015) was the first to report on beliefs in STIs in Madagascar, finding that almost 25% of university students did not believe that HIV/AIDS was a real disease. In Madagascar, STI prevalence rates are higher in young adults than in other age classes. Malagasy women aged 15–24 are over three times as likely to test positive for chlamydia (*Chlamydia trachomatis*) and over seven times as likely to test positive for gonorrhea (*Neisseria gonorrhoeae*) than women aged 25–34 (Leutscher et al., 2005). Behavioral risk factors, such as having multiple partners and infrequent use of condoms, put this age group at high risk of STI infection (Leutscher et al., 2005).

#### The United States of America

The United States has a primarily ‘Western’ culture. Most of the population identifies as Christian, although this number has been shrinking, especially among young people (Pew Research Center, 2015). The United States ranks higher than Madagascar in gender equality (28th and 74th, respectively) and social progress (16th and 124th, respectively) (World Economic Form, 2016). However, compared to many western European countries, the United States’ culture is more conservative and less open to discussions around sexuality, which may contribute to poorer sexual health outcomes (Dodge et al., 2005). Compared to Madagascar, the United States is a wealthier nation (the United States ranks 8th on the Human Development Index; Madagascar ranks 154th; (UNDP, 2015) and has a more robust health system (the United States ranks 37th in terms of overall health system performance; Madagascar was ranked 159th; WHO, 2000). However, both the United States and Madagascar continue to face public health burdens from STIs, including HIV/AIDS (CDC, 2012; WHO, 2013).

In the United States, many studies have looked at knowledge, belief, and disease risk around STIs and HIV/AIDS for young adults. First, studies testing young adults’ knowledge of STIs and HIV/AIDS found that although American young adults score higher than young adults from other countries (Benotsch et al., 2004), they still have significant gaps in knowledge (Hingson, Strunin & Berlin, 1990; Benotsch et al., 2004; Duncan et al., 2005). For example, American STI clinic patients scored higher on an HIV/AIDS knowledge test than Russian clinic patients, but still had knowledge deficits related to the link between STIs and use of preventative methods (differences were unrelated to patients’ educational achievements; Benotsch et al., 2004). Second, few studies have investigated beliefs surrounding STIs and HIV/AIDS among American young adults, although one study found that African–Americans may express higher levels of disbelief in HIV/AIDS (Bird & Bogart, 2005). Finally, in terms of disease risk, young adults (15–24 years) account for half of all newly acquired STIs in the United States and made up the largest percentage of gonorrhea and chlamydia cases reported in 2013 (aged 20–24 years; (CDC, 2013).

## Methods

### Data collection

The study population included university-aged students at one university in the southern state of Florida in the United States of America (USA) and one university in northern Madagascar. Data were collected via in-person surveys distributed to university-aged adults on the campus of the University of Antsiranana (Madagascar, June 2013; see Reuter, Randell & Reuter, 2015) and the Florida Gulf Coast University (United States of America, January/February 2015). Complete survey materials can be found in the supplementary materials (Appendix S1); questions directly relevant to this paper are found in Table 1. We acknowledge that the answer choices may not reflect all possible answers; respondents were verbally asked to pick the answer that most represented their personal situation.

**Table 1:** Selected survey questions and possible responses. Full survey materials and informed consent statement can be found in Appendix S1. Questions listed in the order seen by respondents but numbers may not match those in Appendix S1 and are listed for reference purposes only.

	Questions	Possible responses
1.	How old are you?	Free response.
2.	What is your gender?	Male/Female/Prefer not to answer.
3.	Do you have any children?	Yes/No/Prefer not to answer
4.	What is your marital status?	Single/Married/Divorced/Widowed/ Prefer not to answer
5.	What is your sexual orientation?	Heterosexual/Homosexual/Bisexual/Asexual/Prefer not to answer^a^
6.	Please fill out the following table to the best of your knowledge. List all sexually transmitted diseases that you know about (including HIV/AIDS).	Free response. Table with columns prompting the respondent to provide: a) name of the sexually transmitted disease; b) do you know anyone who has this disease?; c) have you ever had this disease?; d) do you believe that this disease exists?; e) why or why not do you think this disease exists?; f) how do you protect against this disease?; and g) do you know any cures for this disease?

**Notes.**

a ‘Transgender’ was added as an additional answer option under ‘sexual orientation’ for the surveys among students in the United States (though we recognize this is a gender identity rather than a sexual orientation). This was not an answer option on the survey in Madagascar and no students selected ‘transgender’ as an answer in the United States.

Surveys were initially developed for the purpose of surveying French-speaking individuals in Madagascar (Reuter, Randell & Reuter, 2015). The design of the survey is detailed in Reuter, Randell & Reuter (2015). In brief, questions were asked following a prior study of university students in Madagascar (Rahamefy et al., 2008). In preparation for data collection in Madagascar, surveys were developed in English and translated into French by a fluent French speaker not otherwise associated with the project (noting that KER is a non-native French speaker). Both English and French versions aimed for simple and concise questions. English and French versions of the survey were submitted to the Institutional Review Board at Florida Gulf Coast University; this review board has translations of non-English documents verified by third parties as part of the review process. Finally, the French-language survey was tested in pilot interviews with three volunteer university students prior to the onset of primary data collection; their feedback was used to improve the survey instrument (see Reuter, Randell & Reuter, 2015 for more details).

Methods for data collection in Madagascar are detailed in Reuter, Randell & Reuter (2015). Methods for data collection in the United States are detailed here, as are any differences in data collection methods between the two study sites. Surveys distributed at the Malagasy university were in French, while surveys distributed at the American university were in English. Face-to-face recruitment was used to identify survey respondents. In Madagascar, active recruitment was used; students were approached in public areas of the university campus during business hours (8:30 AM–2:30 PM) where the topic, purpose, and risks of the survey were explained by a two-person survey team (one American researcher and a Malagasy/French translator). In the USA, passive recruitment was used; an information booth was set up in a public area of the university campus during business hours (10:00 AM–2:00 PM) where the topic, purpose, and risks of the survey were explained by two-person survey teams (volunteer research assistants). Survey teams provided explanations of the contents of the survey if respondents required clarification. Once respondents elected to participate in the study, they were asked to complete the survey by hand and to provide written consent by checking a box next to a statement indicating that they consented to participate in the research. Respondents could complete the survey in a private location and were reminded that questions could remain unanswered and that participation was voluntary. Upon completion of the survey, respondents were asked to fold the one-page survey paper twice and place it into a container. There were no incentives offered to students to participate in either country.

### Ethical research statement

Research protocols were approved by an ethical review board (Institutional Review Board, Florida Gulf Coast University) prior to data collection (Protocol ID Numbers 2013-32 and 2014-62). All researchers were trained in ethical data collection through the Collaborative Institutional Training Initiative (CITI). Data collection followed all laws relevant to the survey of adult populations.

### Data analysis

Statements made in a foreign language in response to open-ended questions were translated back into English by KER with the assistance of a French-speaking Malagasy assistant who provided assistance during data collection in Madagascar. For questions with categorical answers, data are presented as percentages of the total respondent pool, or a portion of the respondent pool. For open-ended questions, variables were extracted from the most common responses and the proportions of respondents providing those answers were analyzed (following Rahamefy et al., 2008; Reuter, Randell & Reuter, 2015). For questions with quantitative answers, data are presented as means with standard deviations. Sample sizes vary due to the voluntary nature of the survey, but are indicated. All statistical analyses were performed using the JMP software program (JMP^®^, Version 13.1; SAS Institute Inc., Cary, NC, USA).

For objective one, study participants were asked to list all STIs they knew of, including HIV/AIDS. STI awareness was summarized by simply counting the number of STIs, aside from HIV/AIDS, that respondents listed in response to question six (Table 1). For objective one, we first used a Generalized Linear Mixed Effects Model (Poisson distribution, Log link function, Maximum Likelihood Estimation Method) to test whether place of study had an effect on the number of STIs listed by students at the American and Malagasy university in response to question six (Table 1; excluding HIV/AIDS). In this test—using respondents as replicates—country (binary variable: United States or Madagascar) was used as the independent variable, number of STIs listed (scale: 0–5; 1.61 (95% CI [0.14]), *n* = 436) was the dependent variable, and gender (binary variable: Male or Female) was used as a random effect. Second, for objective one, a Wald Chi-Square Test (with gender as a random effect) was used to test whether place of study had an effect on the proportion of students at the American and Malagasy university that listed at least one STI in response to Question 6 (Table 1, excluding HIV/AIDS). In contrast to the Standard Least Squares Mixed Effects Model used, above, a Wald Test has a binary dependent variable; in this case, using respondents as replicates—country (binary variable: United States or Madagascar) was used as the independent variable, whether or not a respondent listed any STIs other than HIV/AIDS (binary variable: STI listed or not) was the dependent variable, and gender (binary variable: Male or Female) was used as a random effect. More information about the Wald Chi-Square Test as it is calculated in the JMP software used for analyses can be found online in the JMP 13.1 online documentation (http://www.jmp.com). Given that the research question aimed to look at the effects of culture on survey responses, and given that gender could have impacted upon surveys responses, gender was used here and elsewhere as a random effect. Here and for other objectives (see below), odds ratios (inclusive of 95% confidence intervals; and test for whether the odds ratio differed from 1) and bivariate proportions comparison tests (Adjusted Wald Two Sample Test for Proportions) were also calculated following the calculation of the Wald Tests.

For objective two, a Wald Test (with gender as a random effect) was used to test whether place of study had an effect on: (1) the belief that gonorrhea was real; (2) the belief that syphilis was real; and (3) the belief that HIV/AIDS was real. Note that analyses for this objective as well as objective three were limited to syphilis, gonorrhea, and HIV/AIDS as these were the only STIs mentioned by at least 20 men and 20 women in both countries (students having been prompted to provide information about HIV/AIDS, Table 1).

For objective three, a Wald Test (with gender as a random effect) was used to test whether place of study had an effect on self-reported rates of STIs. The same tests were then repeated for gonorrhea and HIV/AIDS. No students at the American university reported having had syphilis, however, and therefore a Likelihood Ratio Test (gender as a random effect) was performed.

A table summarizing the generalized linear mixed effects model, the Wald Tests, and the Likelihood Ratio Test, can be found in Appendix S2.

## Results

### Dataset parameters

In Madagascar, 261 surveys were distributed and 242 (92.7%) were returned with written consent. In the United States, all 199 distributed surveys were returned with written consent. Some respondent demographics differed by country; dataset parameters summarized here (Table 2) are expanded upon in Appendix  S3. More females than males were surveyed in America; the opposite was true in Madagascar. Respondents at the American university were younger than respondents at the Malagasy university and less likely to have children. Most respondents, regardless of place of study, identified as single and heterosexual (Appendix  S3). Other respondent characteristics were not collected in our survey (Appendix S1).

**Table 2:** Parameters of the respondent pool. Age is illustrated as the mean ± standard deviation while all other parameters are shown as a percentage of the respondent pool.

	USA			Madagascar		
	All	Female	Male	All	Female	Male
Sample size	199	114	80	242	67	173
Age	21 ± 4	21 ± 5	21 ± 3	23 ± 3	22 ± 4	23 ± 3
Children						
Yes	3.0%	4.4%	1.3%	11.6%	10.5%	12.1%
PNTA^a^	0.0%	0.0%	0.0%	1.7%	1.5%	1.2%
Marital status						
Single	96.0%	94.7%	97.5%	94.6%	94.0%	94.8%
Married	3.0%	4.4%	1.3%	2.9%	4.5%	2.3%
Divorced	1.0%	0.9%	1.3%	0.5%	1.5%	0.0%
PNTA^a^	0.0%	0.0%	0.0%	2.1%	0.0%	2.9%
Sexual orientation						
Heterosexual	77.8%	73.7%	86.3%	86.8%	83.6%	87.9%
Homosexual	5.6%	5.3%	6.3%	3.3%	1.5%	4.1%
Bisexual	11.1%	14.9%	5.0%	0.8%	1.5%	0.6%
Transgender	0.0%	0.9%	0.0%	0.0%	0.0%	0.0%
Asexual	2.0%	3.5%	0.0%	0.0%	0.0%	0.0%
PNTA^a^	3.5%	2.6%	2.5%	9.1%	13.4%	7.5%

**Notes.**

a PTNA, Prefer Not To Answer.

### Culture and awareness of STIs (objective one)

All STIs listed by respondents in response to question six can be found in Table 3. There was mixed support for hypothesis (1). Excluding information provided by students on HIV/AIDS, students at the American university listed 2.17 ± 1.72 (mean ± st. dev) different STIs while students at the Malagasy university listed 1.15 ± 0.99 different STIs; place of study had an effect on the number of different STIs that students listed (Generalized Linear Mixed Effects Model (Poisson distribution, Log link function, Maximum Likelihood Estimation Method), L-R Chi-Square = 69.265, *n* = 436, *p* < 0.0001, gender as random effect). However, when using a different test, which categorized students as either having provided information about an STI (excluding HIV/AIDS) or not, there was no difference in the proportion of students listing at least one STI (70.0% vs. 74.0% of students in the United States and Madagascar, respectively; Adjusted Wald Two Sample Test for Proportions, p = 0.3640), and there was no effect of place of study once adjusting on gender (Wald Test, Chi-Square = 0.843, *n* = 436, *p* = 0.3587, gender as random effect; Odds Ratio (United States/Madagascar) = 0.82, 95% CI [0.54–1.25], *p* = 0.3575).

**Table 3:** Awareness of STIs in the two places of study, including the sample sizes for people who reported information about different sexually transmitted infections (STIs), and the mean number of STIs (±st. dev) listed by respondents in response to question six (Table 1). Note that students were prompted to provide responses regarding HIV/AIDS (Table 1). These totals include individuals that did not specify a gender as part of their survey responses (*n* = 2 people in Madagascar and *n* = 4 people in the United States); these individuals were, however, excluded from any analyses where gender was a random effect.

Sexually transmitted infections (STIs)	USA (*n* = 199 students)	Madagascar (*n* = 242 students)
STIs	Sample sizes (percentage of respondents that listed STI)	Sample sizes (percentage of respondents that listed STI)
Chlamydia	82 (41.2%)	4 (1.7%)
Gonorrhea	91 (45.7%)	97 (40.1%)
Hepatitis^a^	8 (4.0%)	9 (3.7%)
Herpes^b^	118 (59.3%)	7 (2.9%)
HIV/AIDS	174 (87.4%)	215 (88.8%)
HPV/genital warts	35 (17.6%)	2 (0.8%)
Lice^c^	26 (13.1%)	1 (0.0%)
Syphilis	58 (29.1%)	148 (61.2%)
Trichomoniasis	5 (2.5%)	4 (1.7%)
	Means ± st. dev (% of respondents in place of study)	Means ± st. dev (% of respondents in place of study)
Mean number of STIs listed by respondents in response to survey question 6	3.1 ± 2.1 (81.1%)	2.2 ± 1.3 (90.4%)
Mean number of STIs listed by respondents excluding HIV/AIDS in response to survey question 6	2.2 ± 1.7 (74.5%)	1.2 ± 1.0 (70.3%)

**Notes.**

a Includes all types of hepatitis viruses.

b Includes genital herpes, dentitial herpes, and any mention of the herpes virus.

c Including any mention of pubic lice and crabs.

Additional evidence, some qualitative, did not seem to support the hypothesis of differing levels of awareness in STIs between the two places of study. Similar percentages of respondents (2.4% and 2.7% students in the United States and Madagascar, respectively) listed medical diagnoses that were not STIs (Appendix S4). In addition, respondents from both universities often reported information about the same types of STIs (Table 3), both referred to STIs using slang terms, and did so at the same frequency (Appendix S4), and both were equally likely to list condoms as a form of protection against STIs (place of study did not seem to have an effect on the proportion of respondents who listed condoms as a form of protection against syphilis, gonorrhea and HIV/AIDS, *p* > 0.12; Appendix S4).

### Culture and students’ belief in STIs (objective two)

**Figure 1:**
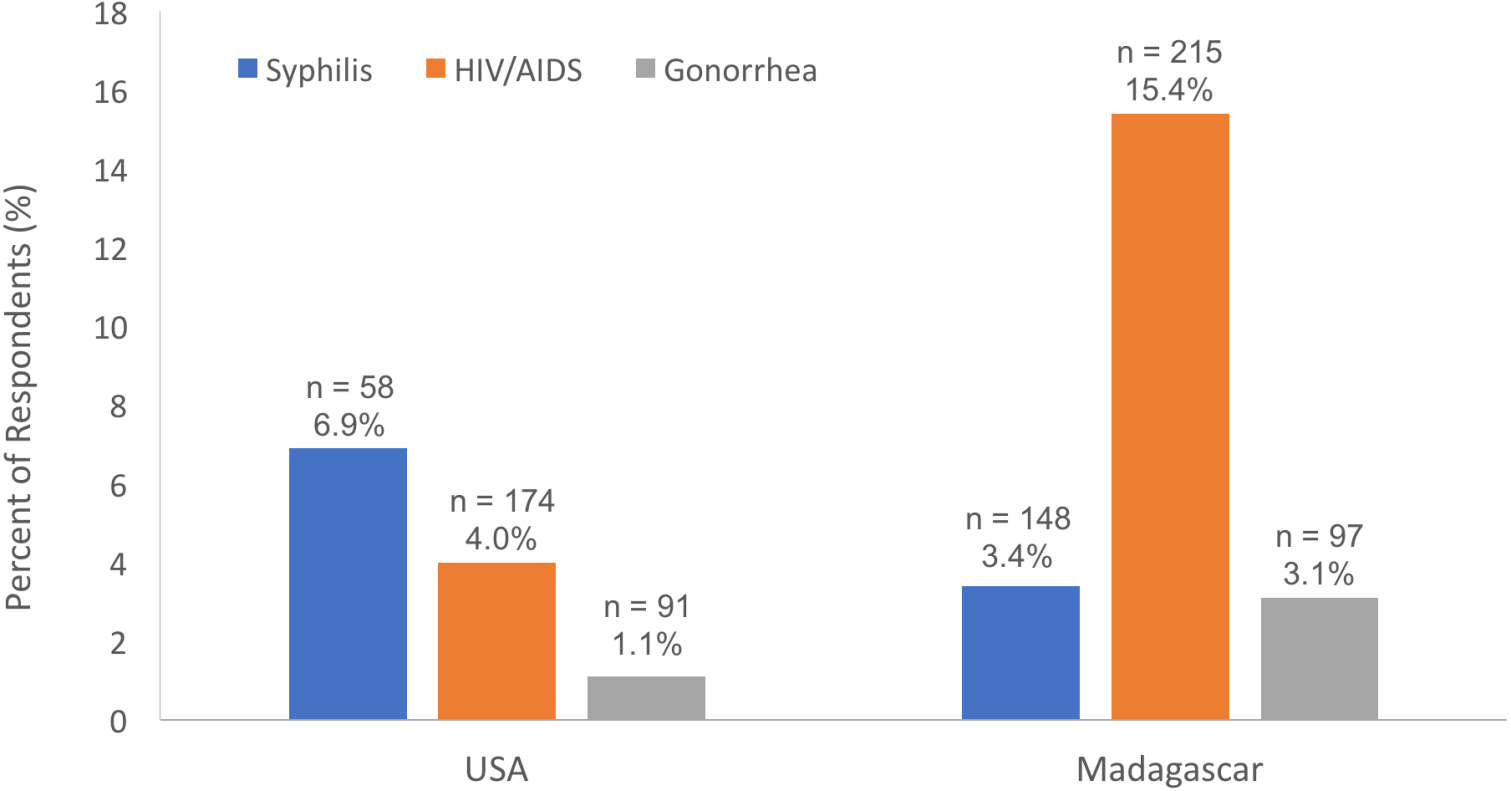
The percent of respondents, by place of study, who expressed disbelief in the existence of common sexually transmitted infections (STIs) (out of the students that provided information for each individual STI). Only STIs with more than 20 respondents for each gender, in each country, are included in the graph.

There was mixed support for hypothesis (2) (Fig. 1). Among students providing information about syphilis in response to question 6 (Table 1), there was no difference in the proportion of students that disbelieved in the existence of syphilis by place of study (3.4% and 6.9% of students in Madagascar and the United States, respectively; Adjusted Wald Two Sample Test for Proportions, *p* = 0.2678; Table 4), and there was no effect of place of study once adjusting on gender (Wald Test, Chi-Square = 1.125, *n* = 182, *p* = 0.2888, gender as random effect; Odds Ratio (United States/Madagascar) = 2.08, 95% CI [0.50–8.19], *p* = 0.2988). Likewise, among students providing information about gonorrhea in response to question 6 (Table 1), there was no difference in the proportion of students that disbelieved in the existence of gonorrhea by place of study (3.1% and 1.1% of students in Madagascar and the United States, respectively; Adjusted Wald Two Sample Test for Proportions, *p* = 0.4471), and there was no effect of place of study once adjusting on gender (Wald Test, Chi-Square = 0.951, *n* = 163, *p* = 0.3296, gender as random effect; Odds Ratio (United States/Madagascar) = 0.32, 95% CI [0.02–2.57], *p* = 0.2949). Among students providing information about HIV/AIDS in response to question 6 (Table 1), however, a higher proportion of students disbelieved in the existence of HIV/AIDS at the Malagasy university, compared to students in America (15.4% and 4.0% of students in Madagascar and the United States, respectively; Adjusted Wald Two Sample Test for Proportions, *p* = 0.0001), and there was an effect of place of study once adjusting on gender (Wald Test, Chi-Square = 11.784, *n* = 353, *p* = 0.0006, gender as random effect; Odds Ratio (United States/Madagascar) = 0.23, 95% CI [0.09–0.50], *p* = 0.0001).

**Table 4:** Awareness, beliefs, and self-reported rates of STIs in the two places of study.

	United States of America (*n* = 199 respondents)			Madagascar (*n* = 242 respondents)		
	Percent of respondents who reported information about this STI (number of respondents)	Percent of respondents who did not believe the disease exists (number of respondents)	Percent of respondents who self-reported having the disease (number of respondents)	Percent of respondents who reported information about this STI (number of respondents)	Percent of respondents who did not believe the disease exists (number of respondents)	Percent of respondents who self-reported having the disease (number of respondents)
Gonorrhea	45.7% (91)	1.1% (1)	3.3% (3)	40.1% (97)	3.1% (3)	12.4% (12)
HIV/AIDS	87.4% (174)	4.0% (7)	0.6% (1)	88.8% (215)	15.4% (33)	1.9% (4)
Syphilis	29.1% (58)	6.9% (4)	0.0% (0)	61.2% (148)	3.4% (5)	17.6% (26)

The three STIs examined here (syphilis, gonorrhea, and HIV/AIDS) were not the only STIs about which students expressed disbelief; two survey participants (one from each place of study) indicated a disbelief in chlamydia and two survey participants in the United States expressed a disbelief in herpes.

**Figure 2:**
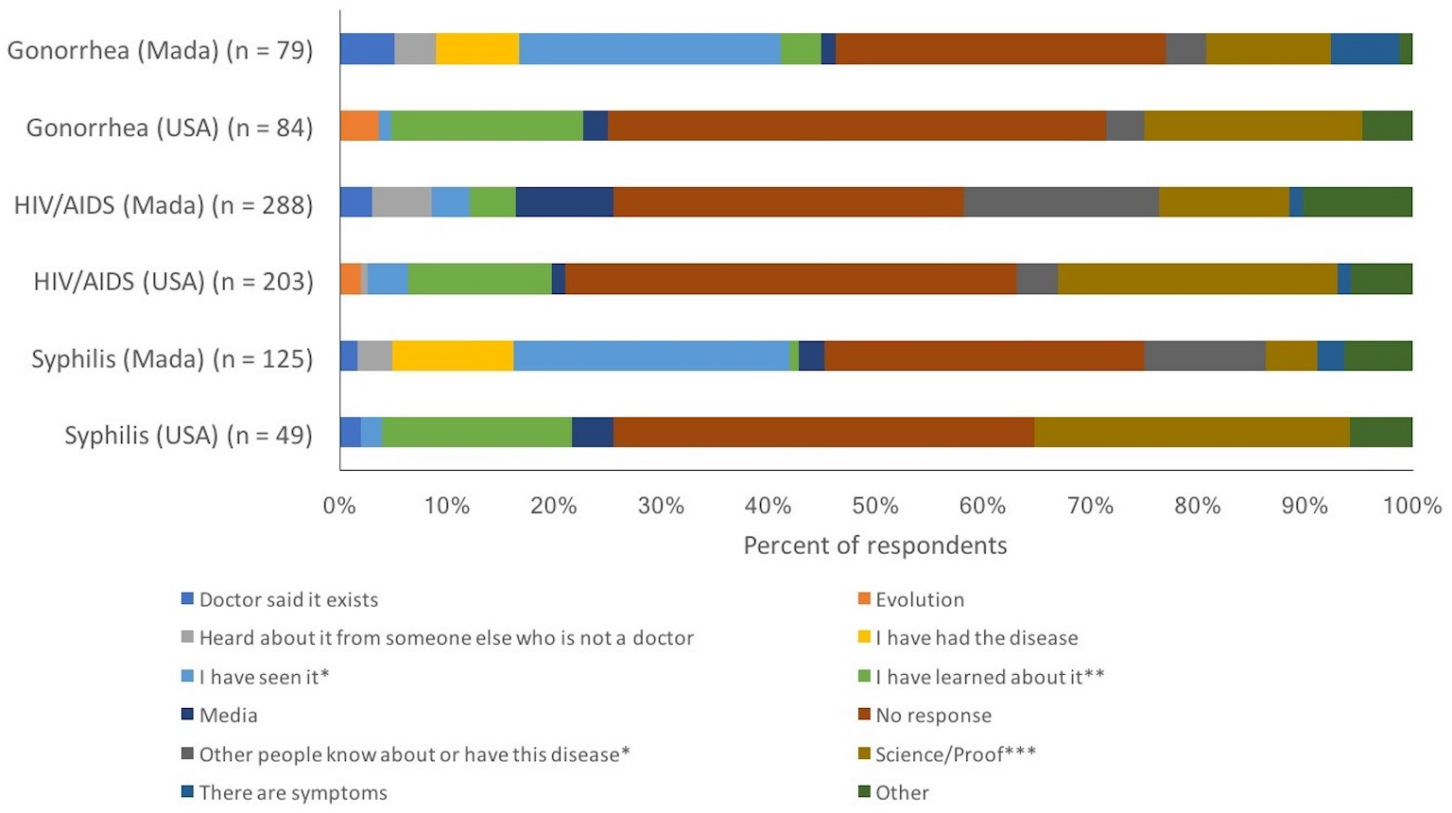
Reasons provided respondents as to why they believed that gonorrhea, HIV/AIDS, and syphilis were real. Sample sizes are the number of people who listed the STI and indicated that they felt it was real. ^∗^ I have seen it/seen people with this illness/know someone with this disease. ^∗∗^ I learned about it/due to outreach/because of the existence of outreach/education. ^∗∗∗^ Science/medicine exists for it/research/statistics/reports/facts/evidence/proof/because it does.

**Figure 3:**
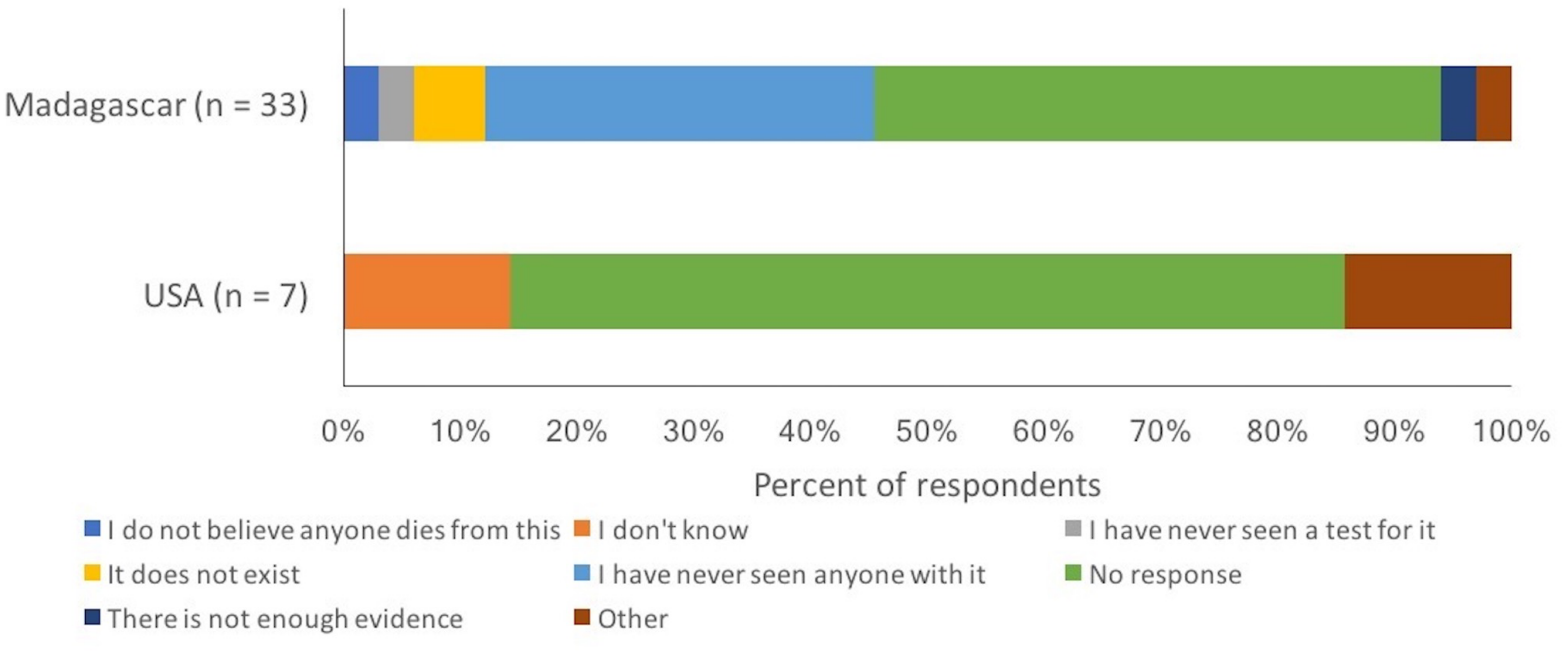
Reasons provided by respondents as to why they felt that HIV/AIDS was not a real disease. Sample sizes were too small to present information for gonorrhea and syphilis.

Students were asked to provide a reason for why or why not they believed that different STIs were real (Figs. 2 and 3). When examining the reasons given for a belief in the existence of STIs from a more qualitative perspective, some interesting observations arise. For both syphilis and gonorrhea, one-third of students at the Malagasy university (32.1% and 37.4%, respectively of students indicating a belief in the disease) referenced either having seen the disease or having had it themselves, as reasons for why the STI existed. In contrast, just one student at the American university (2.0% and 1.2% for syphilis and gonorrhea, respectively) cited these two reasons. For all three STIs, students at the American university often stated that they had learned about these STIs (as reason for their belief in these STIs; 9.1–21.7% of students in the United States, depending on the STI, compared to 1.6–7.4% of students in Madagascar). Students at the American university also often referenced science, medicine, and other forms of proof (15.2–41.9% of the time, when providing a reason for belief in an STI; compared to 6.2–20.0% of students in Madagascar). When students stated why they did not believe in the existence of HIV/AIDS, there were also some interesting observations (Fig. 3). Students in Madagascar explicitly mentioned not having seen various forms of evidence to support the existence of HIV/AIDS. In contrast, students in the United States often did not provide a reason at all to justify their disbelief in HIV/AIDS.

Students at the American university frequently wrote brief statements in response to the question asking why they thought the STI was real, such as “because it does” (*n* = 9), “because it exists” (*n* = 5), “because science” (*n* = 6), or even simply one word statements like “evidence” (*n* = 16) or “science” (*n* = 66). “Sex ed” (short for ‘sex education’, which is typically included in K-12 curriculums in the United States) was also mentioned several times (*n* = 25). Students in the United States rarely provided longer or more insightful responses as to why they believed an STI was real.

In Madagascar, students had a tendency to write more detailed explanations for why they felt STIs were real; they often provided specific anecdotes rather than more general statements. For example, “a lot of people urinating outside seem to have pain while doing so” (*n* = 1), “because my doctor says it exists, so now I truly think it exists” (*n* = 1), “I believed once my friend told me who works in a hospital” (*n* = 1), “I think it exists because the state invests a lot of money to prevent it” (*n* = 1), or “it has been announced using technology, that this exists in our country” (*n* = 1). Like in the United States, a few students also simply provided brief statements like, “because it exists” (*n* = 1), “I believe in science” (*n* = 3), “because science has proven it” (*n* = 2), “it is established in medicine” (*n* = 5), and “it is established in science” (*n* = 1).

In Madagascar, but not the USA, some students referenced non-heterosexual sex or other behavior such as risky sex (i.e., sex without a condom) as evidence for why they believed an STI existed. For example: “after it emerged in gay people, it spread across the world” (*n* = 1), “because there are many people having bizarre/weird sex” (*n* = 1), or “happened because people had sex with animals” (*n* = 1).

### Culture and the proportion of students with self-reported STIs (objective three)

**Figure 4:**
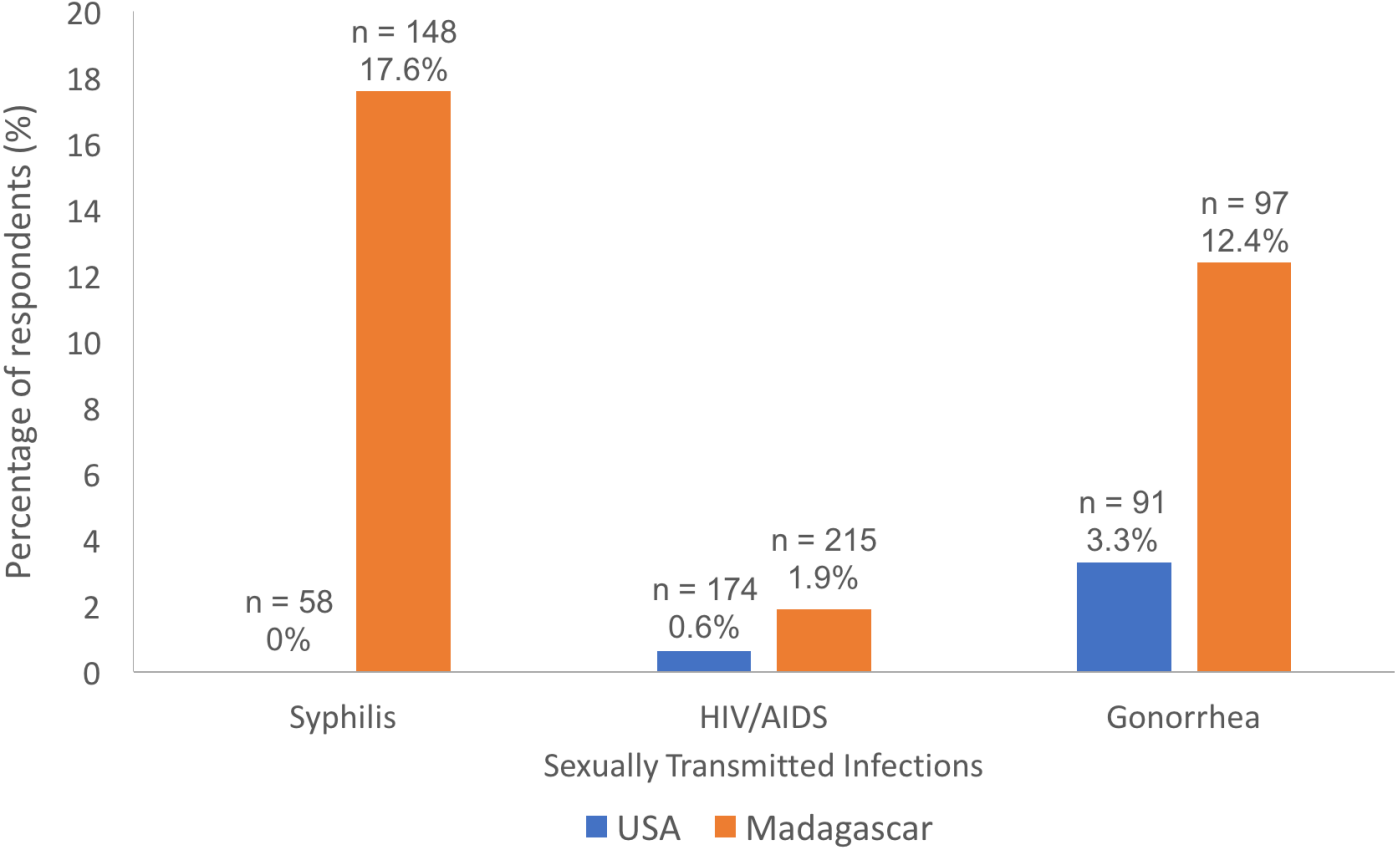
Rates of sexually transmitted infections (STIs) as self-reported by respondents (out of the students who provided information about these STIs in response to question 6; Table 1). It is possible that students who had contracted the STI may not have listed it on the survey and would therefore not have self-reported it to researchers.

In accordance with hypothesis (3), among students providing information about STIs in response to question 6 (Table 1), a higher percentage of students in Madagascar (14.6%) than in the USA (5.1%) reported having had, either currently or in the past, one of the STIs that they named in the survey (Adjusted Wald Two Sample Test for Proportions, *p* = 0.0009). The effect of place of study remained significant once adjusting on gender (Wald Test, Chi-Square = 9.618, *n* = 436, *p* = 0.0019, gender as random effect; Odds Ratio (United States/Madagascar) = 3.18, 95% CI [1.59–6.93], *p* = 0.0008). Specifically, among students providing information about syphilis in response to question 6 (Table 1), a higher proportion of students self-reported having been infected with syphilis in Madagascar (18.8%) than the United States (0%; Adjusted Wald Two Sample Test for Proportions, *p* < 0.0001), with the effect of place of study remaining significant once adjusting on gender (Likelihood Ratio Test, Chi-Square = 19.2988, *n* = 194, *p* < 0.0001, gender as random effect). Likewise, among students providing information about gonorrhea in response to question 6 (Table 1), a higher proportion of students self-reported having been infected with gonorrhea in Madagascar (13.8%) than the United States (3.6%; Adjusted Wald Two Sample Test for Proportions, *p* = 0.023), with the effect of place of study remaining significant once adjusting on gender (Wald Test, Chi-Square = 4.840, *n* = 163, *p* = 0.0278, gender as random effect; Odds Ratio (United States/Madagascar) = 4.32, 95% CI [1.31–19.49], *p* = 0.0146). In contrast, among students providing information about HIV/AIDS in response to question 6 (Table 1), the proportion of respondents that self-reported having been infected with HIV/AIDS did not differ by place of study (2.0% and 0.6% of students at the Malagasy and American universities, respectively; Adjusted Wald Two Sample Test for Proportions, *p* = 0.3840), and no effect of place of study once adjusting on gender (Wald Test, Chi-Square = 1.143, *n* = 362, *p* = 0.285, gender as random effect; Odds Ratio (United States/Madagascar) = 3.32, 95% CI [0.49–65.31], *p* = 0.2374; Fig. 4).

## Discussion

Our study aimed to increase understanding of the influence of cultural setting on young adults’ sexual health by conducting a cross-cultural survey of university students in two different places of study. Using qualitative and quantitative data, we did not find clear evidence that students in either the United States or Madagascar had higher awareness of STIs, but we did find lower rates of belief in HIV/AIDS among students in Madagascar. In addition, students at the Malagasy university were more likely than students at the American university to report having had an STI in the past (specifically syphilis and gonorrhea, but not HIV/AIDS).

### Culture and awareness of STIs

Understanding young adults’ awareness of STIs and their transmission is important for gauging the need for educational campaigns and outreach programs. While it is clear that other factors, including fear of infection (in Brazil; Almedia et al., 2014) may also impact a person’s likelihood to engage in risky behaviors (Fisher & Fisher, 1992), increased knowledge is a known prerequisite for behavior change (though not a predictor, Hingson, Strunin & Berlin, 1990; MacDonald et al., 1990; Fisher & Fisher, 1992; Keller et al., 1991; Diclemente, 1991; Onah et al., 2004).

While we found that the students at the American university listed more STIs than students at the Malagasy university in response to our survey, students in both countries listed medical conditions that were not STIs, referred to STIs using slang terms, and listed condoms as a form of protection against STIs. This may mean that both students in Madagascar and in the United States have a high general awareness of STIs and their prevention, but that students in the United States have been exposed to disease-specific information on a wider range of STIs. As per the more qualitative data presented in the results, students at the Malagasy university seemed less likely to have been exposed to formal information about STIs and more often listed information about STIs that they, or friends, had personally contracted. However, in Madagascar, students did often reference other individuals (i.e., doctors, healthcare professionals, friends whose opinions they valued) as the sources of information for why or why not they believed in the existence of an STI. As such, outreach campaigns in Madagascar could build upon the relatively high level of basic STI awareness (i.e., the high level of knowledge regarding condom use) and the status of healthcare professionals as trusted information sources, to distribute STI-specific information. This might be useful in the case of STIs that do not immediately present symptoms or where the symptoms might be confused for one of the commonly cited STIs in Madagascar.

Our findings on the effect, or a lack of effect, of the place of study on students’ awareness of STIs may be influenced by several factors. For example, previous studies have also found high levels of HIV/AIDS knowledge in Madagascar, but little knowledge of how HIV is spread and how condoms can prevent transmission (Lanouette et al., 2003). For this reason, asking questions about the existence of the disease only, may not present an accurate picture of disease awareness. Further, targeting university students may have biased results; the relatively high education rates of both groups of respondents could have masked whatever differences in STI knowledge might exist between general populations in Madagascar and the United States. For example, a previous study done in Madagascar found that wealthier and more educated women exhibited higher knowledge of the methods of HIV prevention and misconceptions surrounding HIV (Glick, Randriamamonjy & Sahn, 2009). It should be noted, though, that other studies have failed to find a correlation between education and STI knowledge in both the United States (access to past STI education; Kurkowski et al., 2012) and Madagascar (education level of respondents; Lanouette et al., 2003). In addition, there are many other ways in which knowledge of STIs can be quantified and captured (e.g., list STIs and ask to select what they heard, identify symptoms of STIs, Anwar et al., 2010); identify primary prevention strategies, Sherman & Bassett, 2007) that were beyond the scope of our survey.

### Culture and belief in the existence of STIs

Disbelief in the existence of a disease could reduce an individual’s perceived risk of infection (e.g., it is a barrier to HIV/AIDS testing and counseling in Mali; Castle, 2003), thereby influencing risk-taking behavior (Caron et al., 1993; Wendt & Solomon, 1995; Lewis, Malow & Ireland, 1997). Therefore, it is important to understand how belief in certain diseases differs between populations.

We found that place of study had an effect on the rates of belief in HIV/AIDS, with fewer students at the Malagasy university believing in the existence of HIV/AIDS compared with students at the American university. A study of AIDS belief in Mali found that highly educated individuals were more skeptical of AIDS, primarily because they were less likely to be in contact with an infected individual (Castle, 2003). Likewise, in this study, our Malagasy respondents tended to be more educated than the general public (Reuter, Randell & Reuter, 2015) and 37% of respondents who did not believe that HIV was real, also said it was because they have never seen anyone with the disease. In the United States, individuals from certain minority groups sometimes express a disbelief that HIV/AIDS is real for various political and historical reasons (Bird & Bogart, 2005). Since our study design did not specifically target any particular segment of the university student population, it is conceivable that our dataset included a reasonably representative sample of the student population and therefore captured some of this disbelief via our sampling design.

Qualitatively, the reasons provided for the lack of belief seemed to differ by place of study as did the length of the answers provided. In Madagascar, students often appeared not to believe in the existence of an STI because of the lack of exposure to someone with the disease. In the United States, those who identified a reason for not believing in a disease used words like “science” “evidence” or “facts” to explain their reasoning. These very different responses suggest that educational campaigns provided in these settings should have different primary messages in order to target the lack of belief among respondents. For example, educational campaigns in the United States may be more scientific in nature while those in Madagascar may be more anecdotal.

### Culture and the proportion of students with STIs

The proportion of survey respondents with STIs may reflect disease risk among young adults in each setting. Place of study appeared to have an effect on the self-reported rates of syphilis and gonorrhea, but not HIV/AIDS. Higher rates of syphilis and gonorrhea among students at the Malagasy university were expected due to studies reporting high rates of STIs in Madagascar (Behets et al., 2001; Lanouette et al., 2003; Leutscher et al., 2003). In contrast, the comparable HIV/AIDS infection rates across the two countries may be reflective of Madagascar’s historically low prevalence compared with other sub-Saharan African countries (perhaps due to its relative isolation, <1% of the population in (Leutscher et al., 2003; Frickmann et al., 2013; UNAIDS, 2013; 116 out of 100,000 people in WHO, 2013). In addition, the state of Florida has higher HIV/AIDS rates than other parts of the United States (CDC, 2012).

### Relationships between awareness, belief, and self-reported STI rates

Our data suggest, albeit not conclusively, that while awareness of STIs are superficially comparable across the two places of study, there are likely differences in the depth and accuracy of students’ awareness (that remain unexplored in this study). For example, in our study over half of respondents (57%) in the United States were listed the herpes virus when prompted to list all STIs known to them, compared to only 3% of respondents in Madagascar (Table 3). The health implications of these differences in awareness are perhaps partially captured in the self-reported rates of STIs (gonorrhea and syphilis), where a decrease in the quality of awareness (not measured here) might presumably be linked to an increase in the rates of STIs.

In addition, it is also clear that there is a link between the belief in STIs and their prevalence in society, though this study helps show that this link is not always intuitive or straightforward. For example, it is logical to hypothesize that students who reported having had an STI would be less likely to express doubt about its existence. However, in our study, the areas of highest self-reported rates of STIs did not always seem to be the areas with the lowest rates of disbelief (e.g., as with HIV/AIDS in Madagascar, Reuter, Randell & Reuter, 2015). Therefore, further research is needed in order to better understand these relationships.

### Limitations of the study

We acknowledge the limitations of this study, which include a small sample size and a lack of replicate surveys across different sites within each country. The limitations of our dataset from Madagascar are described in detail in Reuter, Randell & Reuter (2015), which concludes that students at the Malagasy university are likely quite different from the Malagasy general public.

Secondly, we acknowledge that collecting additional socioeconomic variables, such as income, religion, nationality, and the number of sex partners would have increased the scope of this study. However, collecting some of these data—such as data related to income—may have decreased the willingness for respondents to participate in the survey, especially in Madagascar. Therefore, we opted to focus the survey on a specific set of questions—many of which had been used in a similar survey of students in Madagascar (Rahamefy et al., 2008) that would limit the length and intensity of the survey.

Finally, it should be noted that the non-random sampling of adults in public areas of the university grounds, both in the USA and in Madagascar, might have introduced selection bias which may be evidenced in the percent of studies that were returned to the research team with written consent. For example, one study in Southern Madagascar found that women in higher education are expected to be caregivers for their extended family (Skjortnes & Zachariassen, 2010). Therefore, adults with children or family obligations—especially women—may have been less likely to spend time on campus between classes, and therefore less likely to have been recruited into our study. In addition, those who self-selected to complete the survey may also feel more comfortable discussing sexual health than young adults in general and may be more engaged campus educational and outreach activities which could have influenced their access to sexual health knowledge.

### Conclusion

Our findings add to the growing understanding of how young people’s knowledge, beliefs, and risk of contracting STIs, including HIV, differ by the culture of a place of study. Although this study did not directly measure more specific knowledge around disease prevention or risky sexual behaviors, a lack of awareness of and belief in STIs or HIV/AIDS would suggest that individuals may not be taking action to protect themselves against these diseases, potentially increasing their risk of contracting these diseases. Further, this study found that place of study had an effect on the beliefs surrounding some STIs, including HIV. For this reason, findings suggest that there is a need to tailor future intervention strategies to the varying needs of young adults in different cultural settings to address these misconceptions and knowledge gaps. However, because most empirically validated HIV/AIDS prevention intervention studies have been conducted in the United States or other developed nations, it is unclear the extent to which these techniques may be usefully adapted for the different conditions (Benotsch et al., 2004). Further research on the relationship between culture and sexual health awareness, beliefs, and behaviors is necessary to sufficiently adapt these programs to young adults in different cultural settings.

##  Supplemental Information

10.7717/peerj.4362/supp-1 Supplemental Information 1 Supplementary Materials Appendices for the manuscript.

## Additional Information and Declarations

### Data Availability

The following information was supplied regarding data availability: The raw data include sensitive information about individuals surveyed (i.e., whether they have contracted STIs in the past). Our research was approved by an Ethical Review Board; this board was informed prior to the start of data collection that we would not share the raw data from the survey with any third parties. This is to protect the respondents of our surveys.
