# Supplementary material for: Comparing the awareness of and beliefs in sexually transmitted infections among university students in Madagascar and the United States of America
Source: PeerJ. 2018 Feb 21;6:e4362. doi: 10.7717/peerj.4362 (PMC5825850; doi:10.7717/peerj.4362)
Supplement: Supplemental Information 1 — Appendices for the manuscript. [file peerj-06-4362-s001.docx]

**Appendix 1: Survey Materials**

**Survey**

- We are conducting an Undergraduate Research Project with the title “Comparison of sexual behavior of students at FGCU and the University of Antsiranana/Madagascar” and would like to anonymously collect information from you.
- Please take your time to read the following information carefully before starting the survey.
- Your participation in the study is completely voluntary. If you decide to participate now you may change your mind and stop at any time, for any reason, without penalty or loss of any future services you may be eligible to receive from the University.
- If you have any questions about this study, you may contact the faculty sponsor Dr. Peter Reuter at 590-7512 or via email at preuter@fgcu.edu.
- If you have any questions about your rights as a subject/participant in this research, or if you feel you have been placed at risk, you can contact the Chair of the Human Subjects’ Institutional Review Board through Sandra Terranova, Office of Research and Sponsored Programs, at 239-590-7522.
- Completing the survey should take no more than 10 minutes.
- You do not have to answer all of the questions in this survey. If you do not feel comfortable answering a specific question, please leave it blank. If you do not feel comfortable taking the survey entirely just deposit the blank survey into the survey box.
- **DO NOT PUT YOUR NAME ON THE SURVEY.**
- **DO NOT SIGN THE SURVEY WITH YOUR NAME.**

**By returning my survey, I agree to participate in this research survey. I understand that if my answers are used there is no one who will be able to identify me.**

**🞏 I agree 🞏 I do not agree**

**___________________________________________________________________________________________**

1. **How old are you? ____________________** years □ Prefer not to answer
2. **What is your gender?** □ Male □ Female □ Prefer not to answer
3. **Do you have any children?**  □ Yes □ No □ Prefer not to answer

**If you have children please provide the following information:**

| Child One: | Age: | Male Female |
| --- | --- | --- |
| Child Two: | Age: | Male Female |
| Child Three: | Age: | Male Female |
| Child Four: | Age: | Male Female |
| Child Five: | Age: | Male Female |

1. **What is your marital status?**  □ Single □ Married □ Divorced □ Widowed □ Prefer not to answer
2. **What is your sexual orientation?** □ Heterosexual □ Homosexual □ Bisexual □Transgender □ Asexual □ Prefer not to answer
3. **Please fill out the following table to the best of your knowledge**:

|  | **Have you participated in this type of sex act?** | **What age did you first do this sex act?** | **How often do you do this sex act per month?** | **Do you use protection when you do this sex act?** | **If you use protection, what kind of protection do you use?** |
| --- | --- | --- | --- | --- | --- |
| **Oral Sex** | □ Yes  □ No  □ Prefer not to answer |  |  |  |  |
| **Intercourse** | □ Yes  □ No  □ Prefer not to answer |  |  |  |  |
| **Anal Sex** | □ Yes  □ No  □ Prefer not to answer |  |  |  |  |

1. **Please fill out the following table to the best of your knowledge. List all sexually transmitted diseases that you know about (including HIV/AIDS)**.

| **Sexually Transmitted Disease** | **Do you know anyone who has this disease?** | **Have you ever had this disease?** | **Do you believe that this disease exists?** | **Why or why not do you think this disease exists?** | **How do you protect against this disease?** | **Do you know any cures to this disease?** |
| --- | --- | --- | --- | --- | --- | --- |
|  | □ Yes  □ No  □ Prefer not to answer | □ Yes  □ No  □ Prefer not to answer | □ Yes  □ No  □ Prefer not to answer |  |  |  |
|  | □ Yes  □ No  □ Prefer not to answer | □ Yes  □ No  □ Prefer not to answer | □ Yes  □ No  □ Prefer not to answer |  |  |  |
|  | □ Yes  □ No  □ Prefer not to answer | □ Yes  □ No  □ Prefer not to answer | □ Yes  □ No  □ Prefer not to answer |  |  |  |
|  | □ Yes  □ No  □ Prefer not to answer | □ Yes  □ No  □ Prefer not to answer | □ Yes  □ No  □ Prefer not to answer |  |  |  |
|  | □ Yes  □ No  □ Prefer not to answer | □ Yes  □ No  □ Prefer not to answer | □ Yes  □ No  □ Prefer not to answer |  |  |  |
|  | □ Yes  □ No  □ Prefer not to answer | □ Yes  □ No  □ Prefer not to answer | □ Yes  □ No  □ Prefer not to answer |  |  |  |

**Appendix 2:**

**Table S1:** The tests used to examine the effects of place of study as summarized from the narrative of the manuscript. Additional tests, including Odds Ratio calculations are described in the results section and are omitted here.

| Objective | Test | Independent variable | Dependent variable | Random effect | Sample size | Chi-square | p-value |
| --- | --- | --- | --- | --- | --- | --- | --- |
| One | Generalized Linear Mixed Effects Model (Poisson Distribution) | Place of study (Madagascar vs. USA) | Number of STIs listed that were not HIV/AIDS (scale: 0 – 5) | Gender (Male or Female) | 436 | 69.265 | < 0.0001 |
| One | Wald Chi-Square Test | Place of study (Madagascar vs. USA) | Whether or not a student listed at least one STI that was not HIV/AIDS (presence/absence) | Gender (Male or Female) | 436 | 0.843 | 0.3587 |
| Two | Wald Chi-Square Test | Place of study (Madagascar vs. USA) | Whether or not a student believed in the existence of syphilis (presence/absence) | Gender (Male or Female) | 182 | 1.125 | 0.2888 |
| Two | Wald Chi-Square Test | Place of study (Madagascar vs. USA) | Whether or not a student believed in the existence of gonorrhea (presence/absence) | Gender (Male or Female) | 163 | 0.3296 | 0.2949 |
| Two | Wald Chi-Square Test | Place of study (Madagascar vs. USA) | Whether or not a student believed in the existence of HIV/AIDS (presence/absence) | Gender (Male or Female) | 353 | 11.784 | 0.0006 |
| Three | Wald Chi-Square Test | Place of study (Madagascar vs. USA) | Whether or not a student reporting having had one of the STIs they named in their survey responses (presence/absence) | Gender (Male or Female) | 436 | 9.618 | 0.0019 |
| Three | Likelihood Ratio Test | Place of study (Madagascar vs. USA) | Whether or not a student reporting having had syphilis (presence/absence) | Gender (Male or Female) | 194 | 19.2988 | < 0.0001 |
| Three | Wald Chi-Square Test | Place of study (Madagascar vs. USA) | Whether or not a student reporting having had gonorrhea (presence/absence) | Gender (Male or Female) | 163 | 4.840 | 0.0278 |
| Three | Wald Chi-Square Test | Place of study (Madagascar vs. USA) | Whether or not a student reporting having had HIV/AIDS (presence/absence) | Gender (Male or Female) | 362 | 1.143 | 0.285 |

**Appendix 3: Respondent characteristics**

Respondent demographics differed by nationality (Table 3). The proportion of females and males surveyed differed between the USA and Madagascar (Wilcoxon Test, *X*^2^ = 43.088, DF = 2, P < 0.0001; Table 3). American respondents were younger than Malagasy respondents (Mixed Effects Model, F_(1, 51.08)_ = 10.6967, P = 0.0019; gender as a random effect) and were less likely to have children (Wilcoxon Test, *X*^2^ = 11.618, DF = 2, P = 0.0030). However, the proportion of individuals who reported different types of marital status (single, married, divorced, widowed) did not differ between the two countries (Wilcoxon Test, DF = 3, *X*^2^ = 4.687, P = 0.1962); most respondents were single (Table S2).

The majority of respondents (> 73% for each gender, in each country; Figure S1) identified as heterosexual. The proportion of respondents identifying with different sexual identities (heterosexual, homosexual, bisexual, and asexual) differed between the US and Madagascar (Wilcoxon Test, *X*^2^ = 37.914, DF = 3, P < 0.0001). The proportion of respondents who identified with different sexual identities did not differ when comparing males and females within the same country (within the US: Wilcoxon Test, *X*^2^ = 8.099, DF = 4, P = 0.088; within Madagascar: *X*^2^ = 3.377, DF = 3, P = 0.3371).

**Figure S1:** The percent of respondents, by gender and by country, that self-identified with different sexual identities.

**Appendix 4: Anecdotal information on the ‘quality of awareness’ of STIs**

In the US survey, 22 students called STIs by a slang term (usually “the clap”) and 4 students mentioned medical diagnoses that are not STIs [e.g., bacterial infections (n = 2); Ebola (n = 1); and yeast infections (n = 1)]. This is equivalent to 13.5% and 2.4% of US respondents who provided information about STIs.

In the Madagascar survey, 20 students used slang terms for STIs (e.g., “*chaude-pisse*”) and 6 students mentioned medical diagnoses that are not STIs [e.g. leprosy (n = 1); loss of sex drive (n = 1); yellow fever (n = 1); tuberculosis (n = 2); and yeast infections (n = 1)]. This is equivalent to 9.1% and 2.7% of Malagasy respondents who provided information about STIs.

Among American respondents, men and women did not differ in the frequency with which they used slang words for STIs (4.48% and 2.86%, respectively; Pearson Chisquare Test, Chisquare = 1.373, DF = 2, P = 0.5032); sample sizes were too small to test whether gender impacted the likelihood of an individual to incorrectly list a non-STI.

Likewise, in Madagascar, men and women did not differ in the frequency with which they used slang words to list STIs (3.31% and 3.21%, respectively; Pearson Chisquare Test, Chisquare = 0.003, DF = 1, P = 0.9529) or in the likelihood of incorrectly identifying a disease as being sexually transmitted (1.65% and 0.00%, respectively; Pearson Chisquare Test, Chisquare = 2.609, DF = 1, P = 0.1063).

**Table S2:** Types of protection suggested by American respondents for common STIs (numbers indicate the number of respondents who named each type of protection). Data for Madagascar can be found in Reuter et al. (2015).

|  | USA | |
| --- | --- | --- |
|  | Females | Males |
| Gonorrhea  Abstinence  Birth control (unspecified)  Condom  Contraceptive (unspecified)  Doesn’t know  Limit fluid exposure  Limit needle use  Limit Sex  Protection  STD check/Testing | 5  1  23  1  0  0  0  1  4  1 | 8  2  14  0  2  0  0  1  1  0 |
| HIV/AIDS  Abstinence  Birth control (unspecified)  Condom  Contraceptive (unspecified)  Doesn’t know  Limit fluid exposure  Limit needle use  Limit Sex  Protection  STD check/Testing | 10  1  42  1  1  3  4  6  5  3 | 9  2  30  0  2  2  0  3  4  0 |
| Syphilis  Abstinence  Birth control (unspecified)  Condom  Contraceptive (unspecified)  Doesn’t know  Limit fluid exposure  Limit needle use  Limit Sex  Protection  STD check/Testing | 4  0  13  0  1  0  0  3  4  2 | 4  0  13  0  1  0  0  1  1  1 |

Protection (not explicitly mentioning condoms): protection/safe sex

Limit sexual contact: monogamy/don’t have sex with infected people/choose your partners/don’t have physical contact/don’t have sex for money/no sex before marriage
Communication: tell people about your infection/ask people about their infection/know sexual history of partner

Limit fluid contact: limit fluid contact/do not have sex doing outbreaks/do not touch contaminated blood.

Testing: testing/get tested

Limit needle use: don’t use needles/don’t share needles

Medication: shots/penicillin/medication/vaccination
